# Occupational Malaria Following Needlestick Injury

**DOI:** 10.3201/eid1010.040277

**Published:** 2004-10

**Authors:** Arnaud P. Tarantola, Anne C. Rachline, Cyril Konto, Sandrine Houzé, Sylvie Lariven, Anika Fichelle, David Ammar, Christiane Sabah-Mondan, Hélène Vrillon, Oliver Bouchaud, Frank Pitard, Elisabeth Bouvet, and Groupe d'Etude des Risques d'Exposition des Soignants aux agents infectieux

**Affiliations:** * The Accidental Blood Exposure Study Task Force (GERES), France;; † Bichat-Claude Bernard University Hospital, Paris, France;; ‡ Hôpital Esquirol, Saint-Maurice, France;; § Hôpital National de Saint-Maurice, Saint Maurice, France

**Keywords:** letter, needlesticks, nurse, occupational accidents, malaria, falciparum, bloodborne pathogens

**To the Editor:** A 24-year-old female nurse was admitted to the emergency room at Bichat University Hospital in Paris, France, on July 4, 2001, with fever, nausea, and general malaise. She had no notable medical history, except spontaneously regressive Schönlein-Henloch purpura at 9 months of age. On admission, after she was given paracetamol, her axillary temperature was 37.6°C. She was slightly jaundiced and reported a mild headache but showed no resistance to head flexion. Her abdomen was depressible but tender. Urinalysis did not show hematuria or signs of urinary infection. Biologic tests indicated normal values except the following: platelets 47.4 x 10^3^/µL, aspartate aminotransferase 307 U/L (normal value <56), alanine aminotransferase 239 U/L (normal value <56), total bilirubin 58 µmol/L (normal value <24), and γ-glutamyl transpeptidase 57 U/L (normal value <35). Results of an abdominal echogram were normal. Result of a blood film to identify *Plasmodium falciparum* was positive for parasitemia at 0.038 per 100 erythrocytes. The patient was given 500 mg of oral quinine three times daily; intravenous quinine was administered 15 hours after admission because she became nauseated. Her malaise persisted for 3 days, but she did not show any signs of malaria. She recovered completely and was discharged on day 6 of hospitalization.

The patient had not traveled outside France except to the United Kingdom years earlier. She did not live near an airport, nor had she been to one recently. She had vacationed in the south of France from June 23 to June 26 but had traveled by car. She had been certified as a registered nurse on May 28 and had been working as a substitute employee at various hospitals in the greater Paris area. On June 21, 2001, she sustained an accidental needlestick injury while taking a blood sample with an 18-gauge, peripheral venous catheter that had no safety feature. She removed the catheter stylet and stuck herself as she crossed her hands to discard the stylet in a sharps container. The needlestick pierced the nurse's glove and caused a deep, blood-letting injury on the anterior aspect of the left wrist. She had no previous history of needlestick injury. She notified the hospital occupational medicine department of her injury on the day it occurred and was given a postexposure interview. In accordance with national postexposure management guidelines, she was tested for HIV and hepatitis C virus (HCV) antibody, and results were negative at baseline; her immunization against hepatitis B virus (HBV) was confirmed. The risk of infection by pathogens other than HBV, HCV, or HIV following a needlestick injury was not discussed during her postexposure interview, and the nurse was not made aware of that risk. The injured nurse did not inform the managing physician that the injury had occurred while she was drawing blood from a patient to determine if the patient was infected with malaria.

By July 1, 10 days after exposure, fatigue, malaise, and fever developed; her temperature was lowered to 38.6°C by taking paracetamol. Her condition returned to normal on July 2 before a second bout of fever and myalgia occurred during the night. She had to leave work early on July 3 because of generalized pain and a temperature of 39°C. The patient's mother is a biologist and was aware that her daughter had sustained a needlestick injury while drawing blood from a patient in whom malaria was suspected. The mother insisted that a blood smear be performed at a private laboratory in Paris. The smear was qualitatively determined positive for *P. vivax*. Subsequently, the patient was admitted to Bichat-Claude Bernard University Hospital with suspected malaria. A repeat blood smear conducted there identified *P. falciparum*.

The source patient was a 28-weeks' pregnant, 30-year-old woman of Kenyan origin who resided in France; she had visited Kenya and returned to France on June 1, 2001. On June 21, she was admitted to the gynecology-obstetrics emergency room at a greater Paris area hospital with fever and malaise. Blood sampling and thin and thick blood smears were performed by the nurse. The source patient's level of parasitemia was estimated at 0.05 per 100 erythrocytes, and oral quinine was initiated. The physician who interviewed the nurse after the needlestick injury verified that the source patient was HIV- and HCV-antibody negative and that the nurse was immunized against HBV. On June 23, although the results of her test for *Plasmodium* were negative, she was transferred to another tertiary care center where IV quinine was administered for nausea and vomiting, and she could be monitored more closely. She recovered fully and was discharged on June 27. Unfortunately, all blood samples or smears from the source patient had been discarded by the time the injured nurse became ill.

*P. falciparum* is a bloodborne pathogen, and malaria is a well-documented complication of transfusion (*1*). Malaria has also been diagnosed after intravenous drug use (*2**,**3*) and breaches in infection control procedures (*4**–**6*), as well as occupational exposures (*1**–**5*). Occupational *P. falciparum* infection after a needlestick injury may be rare; however, such an injury can be potentially severe in nonimmune healthcare workers in countries where malaria is not endemic, especially if the occupationally infected person is pregnant. This situation may also become more common as malaria spreads and as increasing international travel brings potential source patients to hospitals in malaria-endemic countries.

HBV, HCV, and HIV are the pathogens most often transmitted in documented cases of occupational infection following needlestick injuries in industrialized countries. Testing for infection by these pathogens does not include all the possible infections that can result from occupational exposure (*1**,**7**,**8*). Although conducting a thorough investigation of the circumstances surrounding any needlestick injury is a challenge in the daily clinical setting, an investigation should always be carried out. As in this case-patient, the treatment of occupational *P. falciparum* infection may be delayed because physicians do not immediately consider malaria as a possible diagnosis. Furthermore, healthcare workers with neurologic symptoms caused by *P. falciparum* malaria may be too ill to tell the treating physician about their occupational exposure. Such infections must be diagnosed promptly as they are potentially lethal, and presumptive treatment is readily available and well tolerated. Clinicians managing healthcare or laboratory workers with a febrile illness or in a postexposure setting should consider the probability of occupational *P. falciparum* malaria.
